# Tuberculosis in migrants – screening, surveillance and ethics

**DOI:** 10.1186/s41479-020-00072-5

**Published:** 2020-09-05

**Authors:** Gabriella Scandurra, Chris Degeling, Paul Douglas, Claudia C. Dobler, Ben Marais

**Affiliations:** 1 Marie Bashir Institute for Infectious Diseases and Biosecurity, The University of Sydney, Sydney, Australia; 2 Australian Centre for Health Engagement Evidence and Values, University of Wollongong, Wollongong, Australia; 3 International Organization for Migration (IOM), Geneva, Switzerland; 4 Institute for Evidenced-Based Healthcare, Bond University, Gold Coast, Australia

**Keywords:** Tuberculosis, TB, Migration, Migrants, Review, Pre-screening, Ethics

## Abstract

Tuberculosis (TB) is the leading infectious cause of human mortality and is responsible for nearly 2 million deaths every year. It is often regarded as a ‘silent killer’ because it predominantly affects the poor and marginalized, and disease outbreaks occur in ‘slow motion’ compared to Ebola or coronavirus 2 (COVID-19). In low incidence countries, TB is predominantly an imported disease and TB control in migrants is pivotal for countries to progress towards TB elimination in accordance with the World Health Organisations (WHO’s) End TB strategy. This review provides a brief overview of the different screening approaches and surveillance processes that are in place in low TB incidence countries. It also includes a detailed discussion of the ethical issues related to TB screening of migrants in these settings and the different interests that need to be balanced. Given recognition that a holistic approach that recognizes and respects basic human rights is required to end TB, the review considers the complexities that require consideration in low-incidence countries that are aiming for TB elimination.

## Introduction

Tuberculosis (TB), is one of the oldest airborne pathogens; found in human remains from nearly 10,000 years ago [1]. Despite its ancient history, TB remains the number one infectious disease killer on the planet (WHO report) with an estimated 10 million people developing TB every year, resulting in nearly 2 million deaths [2]. TB disproportionally affects socioeconomically disadvantaged populations living in low-and middle-income countries in Asia and Africa and is often referred to as the “silent epidemic”, because of its relatively slow spread and the fact that affected populations are often ‘less visible’. *Mycobacterium tuberculosis*, the causative organism of TB disease, is approximately 50 times slower to grow than other common bacteria, and TB disease develops more slowly than high profile viral epidemics such as Ebola or coronavirus 2 (COVID-19). Because TB mainly affects socioeconomically disadvantaged groups [3] (whose voice is often not heard) and appears less threatening to high-income countries than other pandemics, research progress on TB diagnostics and treatments has been slow.

Galvanized by the World Health Organization’s STOP TB and END TB campaigns [4, 5], active case finding initiatives have successfully reduced the prevalence of TB [6, 7] and randomized control trials are informing better ways to find and treat TB cases [8, 9]. The importance of new tools, and better ways of finding and treating all people with TB are well recognized, as is the need to address the many social, financial, ethical, and cultural barriers that limit access to TB prevention, diagnosis and care [2]. But progress requires high level political commitment and sufficient financial resources to implement solutions that have been shown to work. A holistic approach is essential to effectively tackle the many challenges posed by global TB control [10], including careful consideration of TB in migrants. Here we focus on the practice and ethics of TB screening and surveillance in migrants.

## A world in motion

Globalization while traditionally focused on trade and finance has become one of the driving factors of people movement around the world. Improvements in transportation technology, decrease in travel time and costs have been integral in changing the patterns of migration in the world today with 3.4% of the world’s population residing in a country other than their county of birth (IOM). Global mobility and connectivity supports growth in international trade, exchange of ideas, new technology and social/cultural interaction, but it also increases the risk of infectious disease spread and global pandemics, as illustrated by the current severe acute respiratory syndrome COVID-19 outbreak.

## Transmission sustains the epidemic

The global TB epidemic, declared by WHO as a global health emergency in 1993, is driven by ongoing transmission [11] and factors increasing population vulnerability [12], which also applies to emerging drug resistant disease [13–15]. Modelling suggests that adolescents and young adults are major drivers of TB transmission in high incidence countries [16] . During the period 2000–2017, the total number of international migrants rose from 173 million to 258 million, an increase of 49% [17].

Of the infected people who develop TB disease, the majority will become ill within 2–5 years from their most recent infection, but in some people TB disease may be many years after the last infection episode [18]. In low TB incidence countries where TB transmission is limited and most latent TB infection [LTBI] (as well as active disease) is imported from high incidence settings [19], preventing re-activation of LTBI is a major TB control strategy in migrant populations [20, 21]. However, given the high mobility and circulatory nature of migrant populations and the fact that the organism moves with people and crosses national borders with ease [22, 23], future re-infection when visiting areas of the world with poor epidemic control where most TB and drug resistant TB (DR-TB) transmission occurs [11], remains a concern.

## Active TB screening

TB screening policies for migrants attempt to protect populations in low incidence countries against imported TB disease and these mainly focus on pre-migration screening of migrants prior to obtaining travel approval, as implemented by Australia, Canada, New Zealand (NZ), the United Kingdom (UK) and the United States of America (US) [24], as well as the Gulf Corporation Countries (GCC) or post-migration screening after migrants arrive in their destination country, which is predominantly the European approach [25]. A systematic review that analysed TB screening policies in 15 countries, demonstrated poor consistency of practice and standardization [26], stemming from the distinct geographies of each receiving country and different migrant entry routes. Variation in TB screening practices among migrants also reflects the lack of evidence to guide the most effective, efficient and cost-effective TB screening approaches.

Variation in national policies and practices for TB screening in migrants mainly relate to differences in risk factor assessment and different risk thresholds based on the estimated TB incidence in the country of origin [27, 28]. Discrepancies include the definition of a “high TB burden country” (ranging from 40, 60 or 100 per 100,000 population) [27, 29], the intended period of stay (usually greater than 3 or 6 months) and the visa category that triggers formal TB screening and post-arrival surveillance of migrants deemed at high risk of developing TB identified during initial screening. People on refugee or humanitarian visa subclasses have often been considered as high-risk groups [28, 30], but their TB risk is highly variable and dependent on the specific refugee cohort (e.g. based on the TB incidence in the country of origin). Practices differ with some countries in Europe (like Greece and Spain) screening all asylum seekers and others (like Italy) not having any formal screening of asylum seekers [31]. Countries like Australia, screen all persons entering on humanitarian visas (which are a permanent visa category) and when determining the TB risk posed by temporary migrants in Australia, great emphasis is placed on the estimated TB incidence in the country of origin and their declared length of stay [32].

There are many barriers to TB screening, detection and treatment among asylum seekers and refugees, one being stigma, and these will be further discussed in the ethics section of this review. Only through data sharing and research will we acquire better evidence to guide optimal TB screening methods in migrants. Access to essential health services by refugees and migrants vary by country [33] and are determined by national law. Implementation of the improvements to migrant and refugee health, must take account of specific country situations and be in accordance with national legislation, priorities and circumstances, and international instruments on equal access to public health care services.

## TB surveillance and monitoring

Electronic TB databases, such as E-detect in Europe [34] and eMedical in Australia [35] have been established for the better surveillance and epidemiological analysis to inform future TB control interventions [36]. E-detect arose from a tuberculosis consortium, shared by 6 European Union countries, Sweeden, Netherlands, UK, Italy, Romaina and Bulgaria, bringing together world leading TB experts in national public health agencies with industry and major academic centres to address the high disease burden of TB and multi-drug resistant TB through evidenced-based interventions, in vulnerable EU populations, including migrants [37]. E-detect is geared towards TB diagnosis and detection among new and settled asylum seekers and refugees and, importantly, linking patients into the health care system once they have been diagnosed. E-detect also provides standardized TB data from partner countries, including data on LTBI, with the goal of enhancing intercountry collaboration to support the implementation of future TB screening policies and frameworks. Participating countries collect, clean and recode data using a unique de-identified identifier on a range of TB screening data fields (country of screening, screening scheme, sex, age, country of origin, screening performed, screening results and where available, data on initiated and completed treatment), which is then uploaded to the shared database [34]. This allows analysis of best practice from countries where national strategic plans have been most effective, aiding the development of shared and optimised frameworks for enhanced TB control.

E-detect can also be linked with the E-Detect TB smartphone App to assist active case finding among contacts and detect co-prevalent cases [38]. Rapid ‘digital screening’ via a mobile device supports the rapid identification and testing of those individuals who require prioritization for sputum collection and Xpert Ultra® testing. Use of this system have been shown to assist earlier diagnosis and the direct transfer of relevant patient information to referral hospitals in Italy, improving the overall cascade of care in vulnerable and marginalized migrant communities [38].

In Australia, the eMedical database of the Department of Home Affairs collects all TB health information of migrating individuals from the health screening process conducted pre- or post-migration [35]. The database has particular value to identify migrants at high risk of developing TB post-arrival, for example if a lung lesion consistent with TB is detected during initial chest radiography screening, but without microbiological proof of active TB disease. These migrants will undergo ongoing surveillance for disease development or receive preventive TB treatment post-arrival once active disease has been ruled out [39]. Migrants at high risk of developing TB enter into a legally binding agreement as a specific condition of a visa to comply with TB surveillance conducted by local TB health services, referred to as a ‘TB undertaking’. Similarly, electronic databases used by Canada, NZ, and the US, collect information including country of origin, date of birth, gender, occupation, visa details, health data including self-declaration on TB symptoms, previous TB contact, results of chest radiographs, diagnostic test results from sputum smear or culture, treatment regimens and medical follow-up from migrants. This information is vital for TB control with the goal to eventually eliminate TB [21, 40, 41].

Whole genome sequencing (WGS) provides excellent *M. tuberculosis* strain resolution in order to differentiate imported from locally transmitted strains and strain clusters [42–46]. Australia’s most populous state New South Wales implemented routine WGS of all culture-confirmed TB cases in 2016, which guided better targeted public health responses and confirmed that local transmission is limited [12, 47, 48]. WGS provides valuable information to assist individual patient management and public health responses, but widespread implementation in resource-limited settings remains unrealistic at present. Enhanced polymerase chain reaction (PCR) based methods may be more feasible in the interim, both for genetic drug resistance determination and transmission tracking [49]. In time, the establishment of global TB surveillance systems will complement epidemiological surveillance and could track cross-border transmission dynamics, but also pose risks to personal freedom that should be considered (see Ethics section).

## LTBI screening

Acknowledging that LTBI provides a reservoir of infection from which future TB cases may arise [50], low-incidence countries have increasingly implemented LTBI screening among migrants, either pre-departure or post arrival [24, 51]. According to a survey of policies in 29 Organisation for Economic Cooperation and Development (OECD) countries, 16 countries (55%) routinely screened migrants for LTBI [52]. Target populations for LTBI screening vary by age and the country of origin. Australia, and the US only screen migrant children (aged up to 10 years for Australia and up to 15 years for US) for LTBI, as a marker of possible TB disease that justifies a chest radiograph if positive. Testing for LTBI is done using a traditional tuberculin skin test (TST) or an interferon-gamma release assay (IGRA), which has higher specificity.

In a Taiwanese study, the highest number of TB cases found among migrants were identified less than 2 years after migration in individuals who had a clear chest radiograph at initial TB screening [53]. In other settings, the documented risk of LTBI reactivation is elevated in migrants who have fibrotic scars ≥2 × 2 cm and/or calcified nodules ≥1.5 mm on chest radiograph consistent with previous TB infection [24, 54, 55]. An Australian study found that 1.3% of migrants (428/32,550 migrants) deemed to be at a high risk of developing TB based on pre-migration screening (mainly because of chest radiograph abnormalities) developed TB [39]. Of these TB cases, 270 (63%) were diagnosed during active TB follow-up in the first 2 years post migration; the period of their ‘TB undertaking’.

Treating high risk migrants for LTBI, once TB disease has been ruled out, should benefit individual migrants and the general public [56], although formal cost effectiveness analyses remain limited. A systematic review of 16 studies on LTBI screening among migrants did not arrive at any definite conclusions about the (cost-) effectiveness of this intervention [57]. The (cost-) effectiveness of LTBI screening and treatment among migrants was limited by the high number of migrants lost at some level of the care cascade and the fact that only a small number completed LTBI treatment. A modelling study showed that pre-migration screening using an IGRA combined with post-arrival treatment with 4 months of rifampicin among migrants from countries with moderate to high TB incidence resulted in the best cost-effectiveness ratios [58].

With increased global mobility, a growing number of short-term migrants enter low TB incidence countries repeatedly without TB screening, because their intended period of stay is less than the specified cut-off triggering such a requirement. It is unclear to what extent these temporary migrants might contribute to TB disease importation, but the benefits of screening and treating these migrants for LTBI would likely be diminished by ongoing TB exposure in their countries of origin. The legitimacy of pre-migration TB screening has been questioned, [59, 60] with some arguing that better primary care should be available for migrants to support their general health and wellbeing, as well as early TB identification. Patient-centered approaches to TB care have been shown to reduce diagnostic delay, with likely reduction in TB transmission, and improve treatment outcome in migrants [61]. We explore some of the tensions between the classic public health and social dimensions of the problem, as well as the resultant ethical dilemmas in greater detail.

## Ethical dimensions

The TB status of migrants has normative dimensions because of the potential health impacts to individuals and others, and the associated limitations a positive diagnosis can have on their movements within and between countries. Ethical issues related to migrant TB screening are briefly discussed and summarised in Table 1. TB screening of migrants should mainly be justified on the basis of providing appropriate medical care [62]. Being diagnosed with TB or LTBI can serve as a point of discrimination, which can impinge upon the opportunities and choices available to individuals, and increase the risk of harms through TB-related health inequities and social stigma. Current conceptualizations of stigma identify two basic forms [63]. ‘Enacted TB stigma’ refers to exclusion, rejection or devaluation by others based on beliefs of social unacceptability. ‘Perceived TB stigma’ refers to patient and family fears of inferiority stemming from the anticipation of an adverse judgment related to a TB diagnosis. Both cause harms to individuals and groups and are recognized to be a significant barrier to health care utilization and adherence to TB treatment [64, 65]. Consequently, as is the case with all other TB patients, the privacy and confidentiality of migrants using TB services needs to be protected at all times [62].

**Table 1:** Overview of ethical issues related to migrant TB screening

Issues identified	Key considerations
**Care obligation linked to TB screening**	The human rights issue of providing appropriate medical care to all people, irrelevant of TB or LTBI
**Stigma**	
Enacted TB stigma	Exclusion, rejection or devaluation by others based on beliefs of inferiority or social unacceptability
Perceived TB stigma	Patient and family fears of inferiority stemming from the anticipation of an adverse judgment related to a TB diagnosis
“At-risk group” stigma	Magnify a migrants sense of being “out of place”. This requires careful attention to appropriate service design and communication strategies. How best to communicate with migrants to inform them about the potential benefits and risks of TB and LTBI screening requires more research.
**Privacy and confidentiality**	Needs to be respected and protected at all times
**Racial vilification**	Irresponsible media reporting of TB in migrants is implicated in the production of stigma and should be avoided; and racial vilification banned/censored on social media
**Health equity**	All migrants (whether they have TB or otherwise) should have access to basic health services that are both medically and culturally appropriate.
**Trust in the health care system/provider**	Migrants come from countries with weak health systems and often mistrust health systems. Building trust is essential to build constructive and respectful relationships between service providers and migrant communities.

*LTBI* Latent tuberculosis infection, *TB* Tuberculosis

Unfortunately, historical TB control efforts frequently entwined immigration and public health law, which exacerbated stigma and xenophobia and requires constant vigilance [66, 67]. Under these conditions TB screening is not politically benign, but has broad consequences for those being targeted to participate. Concerns about the public health aspects of migration processes increasing the risk of racial vilification are not insignificant, as several studies suggest that TB control measures and representations of migrants in the media as agents of disease may exacerbate stigma [68]. A systematic review of qualitative studies indicates feelings of stigmatization derived from being labelled as a member of an “at-risk group” for TB by health services in their new country of residence, can further magnify migrants sense of being “out of place” [69].

Little research has focused on the specific experience of new migrants, nor sought their views on the best ways forward. For those organizing TB programmes and migration processes, key decisions need to be made as to the most appropriate setting for migration related TB screening and how best to communicate with migrant groups about the potential benefits and risks of these activities [70]. Whether TB screening is conducted pre-migration, as a mandatory part of standard immigration processes, or provided as a non-mandated service, before or after arrival in the new country, distributes the burdens of testing and TB diagnosis differently. Respect for human rights means that participation in health screening should be voluntary – to the extent that competent individuals should always be acting autonomously on the basis of their informed preferences. Nevertheless, some forms of coercion can be ethically justified if they promote significant benefits for the wider population [71, 72]. Because earlier TB case identification will reduce the future population burden of TB disease, screening that is linked to entry or legalizing residence can achieve this aim by potentially identifying and treating immigrants with TB at the earliest time point to prevent transmission within the recipient/host country. However, the use of these mechanisms to enforce participation and potentially transfer screening costs should be carefully considered to minimize harm and creates responsibilities for immigration authorities.

Because access to health care in countries of origin may be limited, it can be challenging for immigration authorities to meet migrants’ health needs if pre-migration treatment is required. Coercion in the context of TB screening can also be counter-productive if it is not accompanied by clear communication about the implications of the diagnosis, the disease or the LTBI detection [73] People will ignore or doubt the importance and purpose of TB screening if the reasons they are given for participation are at odds with their worldviews, values, or both [74]. Poor communication and a failure to accommodate the values of those being targeted can lead to non-compliance and subsequent treatment failure. When treatment is undertaken in the recipient/host country, uncertainty about an individual’s legal status may exacerbate problems of health care access [65]. Challenges faced by migrants such as communication problems, loss of social support, discrimination and acculturation can be aggravated by stigma and other socio-cultural impacts of a positive TB diagnosis [75]. Nevertheless, because of health equity, social justice and public health concerns, it is important that all migrants (whether they have TB or otherwise) have access to basic health services that are both medically and culturally appropriate [76, 77].

As outlined above, the implementation of TB screening often results in conflicting interests and creates a tension between the obligation of the state to protect the health of the public on one hand, and respecting the autonomy, health interests, and liberty of the screened individuals on the other. Screening also creates obligations on the screener to ensure that participants are properly supported and have pathways to address detected abnormalities [78]. Screening should only be undertaken with the intention to treat and with an appropriate level of counselling ahead of such screening. The principle of reciprocity is important to guide the inclusion of certain ‘migrant rights’ into screening guidelines. Screening creates a two-way obligation, upon migrants to actively participate in the programme, and upon authorities to reciprocate the migrants’ participation and the benefits for the control of public health [79].

In considering these background conditions, trust is an important requirement for appropriate service design and delivery for TB screening. Trust is also central to effective communication because it can initiate or build constructive and respectful relationships between service providers and migrant communities. Migrants often come from countries with weak health systems so may not trust providers or be encultured to seek professional health advice [65]. To engender trustworthiness, there is a need for clarity from service providers as to whom any migration-related screening programme is designed to benefit and the rationale underpinning decisions about how resulting burdens are distributed [78]. When considering the benefits and burdens of TB screening, the potential for stigma must be a key concern. TB stigma commonly arises from public health responses to TB in ways that influence affected populations as well as relevant institutions [80–83]. This is not to say that manifestations of stigma are inevitable; careful attention to appropriate service design and communication strategies can work to mitigate these impacts and risks [74]. Systematic evaluations indicate that the optimum approach in high-migrant receiving countries is to offer screening in a range of settings, incorporating a strong focus on community engagement and partnership with migrant organisations in both the design and implementation of screening approaches [65, 75]. Such programmes should ensure that access to diagnostics and treatment are free of charge to maximise access and adherence.

## Conclusion

Aiming for TB elimination in countries where TB is essentially an imported disease requires careful consideration of migrant populations’ best interests and what a holistic TB response should look like. Particular areas requiring better evidence-based solutions include 1) objective and pragmatic risk stratification approaches, 2) better screening and diagnostic tools, and 3) optimal management of LTBI in situations where disease risk is low or the risk of future re-infection high [84, 85]. Given near-total travel bans implemented in the wake of the COVID-19 epidemic, it is an opportune time to critically reconsider and refine migration policies as borders begin to re-open.

## Data Availability

Not applicable.

## Abbreviations

TB: Tuberculosis
DR-TB: Drug resistant TB
LTBI: Latent tuberculosis infection
IOM: International organisation for migration
WHO: World health organisation
TST: Tuberculin skin test
IGRA: Interferon-gamma release assay
COVID-19: Coronavirus 2
NZ: New Zealand
UK: United Kingdom
US: United States of America
GCC: Gulf Corporation Countries
WGS: Whole genome sequencing
PCR: Polymerase chain reaction
OECD: Organisation for Economic Cooperation and Development
